# Detection of urinary *Chlamydia trachomatis*, *Mycoplasma genitalium* and human papilloma virus in the first trimester of pregnancy by PCR method

**DOI:** 10.1186/s12941-018-0276-7

**Published:** 2018-06-04

**Authors:** Monireh Rahimkhani, A. Mordadi, M. Gilanpour

**Affiliations:** 1 Department of Lab Medical Sciences, Faculty of Allied Medical Sciences, Tehran University of Medical Sciences, Tehran, Iran; 2 Department of Epidemiology, Pasteur Institute, Tehran, Iran; 3 Almahdi Kheyrieh Clinics, Tehran, Iran

**Keywords:** Pregnant women, Miscarriage, *Chlamydia trachomatis*, *Mycoplasma genitalium*, Human papilloma virus, Urine sample

## Abstract

**Background:**

Miscarriage and preterm delivery are the most important challenges of pregnancy. Different bacterial and viral infection may cause miscarriage and preterm delivery. Among bacterial factors, *Mycoplasma genitalium* and *Chlamydia trachomatis* have the most important role and human papilloma virus (HPV) is the leading viral factor in this regard.

**Methods:**

First void urine samples were collected from 119 pregnant women who visited health centers for routine first-trimester screening (12–14 weeks gestation). About 10 ml of the sample was centrifuged at 3000×*g* for 20 min and 1–2 ml of the sediment was transferred to sterile microfuges and stored at − 20 °C until analysis. DNA extraction was conducted using A101211 kits imported by Pars Tous Biotechnology Company. The following commercial kits, imported by Pars Tous Biotechnology, were used for PCR.

**Results:**

There is no significant association between urinary isolation of *C. trachomatis* and miscarriage (P = 0.93) and there is no significant association between urinary isolation of *M. genitalium* and miscarriage (P = 0.80). Regarding HPV, since all urine samples were PCR-negative, comparison was not possible. *C. trachomatis* was isolated from the urine samples of 6.72% of the pregnant women who underwent first-trimester screening in health centers using PCR. Previous studies reported a mean chlamydia isolation rate of 3% from urine specimens collected from pregnant women in general. *T* test showed no significant difference between the two groups (P = 0.10). Based on present study the mycoplasma isolation rate was 17.65% using PCR. Previous studies reported a mean mycoplasma isolation rate of 10% from urine specimens collected from pregnant women in general. T-test showed a significant difference between the two groups (P = 0.03).

**Discussion:**

First void urine samples in pregnant women may be an appropriate sample for detection of *C. trachomatis* and *M. genitalium*; however, it is not a good method for HPV isolation therefore vaginal or cervical discharge specimens should be used instead for detection of HPV.

## Importance

In the present study, we decided to investigate three major causes of STDs that also cause miscarriage in the first trimester in a number of pregnant women. Since cervical swabbing is invasive and dangerous and is not accepted by pregnant women, first void urine specimens were used in this study. Among bacterial agents, *Chlamydia trachomatis* and *Mycoplasma genitalium*, and among viral agents, HPV, have a major role in miscarriage. These agents were surveyed in present research.

## Background

Miscarriage is one of the greatest risks threatening the life of the mother and fetus. Different bacterial, viral, and parasitic factors can cause miscarriage. Statistics suggest that more than 90% of miscarriages are related to an infectious or inflammatory factor in the placenta or amniotic fluid [1]. Among bacterial agents, *Chlamydia trachomatis* and *Mycoplasma genitalium*, and among viral agents, human papilloma virus (HPV), have a major role in miscarriage.

*Chlamydia trachomatis* is an obligate intracellular, nonmotile, Gram-negative bacterium in the family Chlamydiaceae. Because it is an intracellular parasite, it does not grow on bacteriologic cultures, and cellular cultures are used for its isolation and reproduction. *Chlamydia trachomatis* is the major cause of trachoma but it can also cause miscarriage, fetal anomaly, endometritis, preterm delivery, and stillbirth. Since this bacterium is not easy to culture and serological methods have a rather low accuracy, molecular methods like PCR are used to detect it.

*Mycoplasma genitalium* is a Gram-negative, pleomorphic, nonmotile bacterium lacking a cell wall. It requires special media for growth and reproduction. It is a leading cause of miscarriage in pregnant women. Since mycoplasmas are difficult to grow and colonies grow after 3–7 days even on special media, it is rarely possible to isolate the bacterium from clinical specimens; therefore, other diagnostic methods are used. One of these methods is serological tests that are not very reliable and false positive results and cross-reaction are frequent in these tests. Therefore, molecular methods like PCR are used for detection. Similar to chlamydia, the samples used to detect mycoplasma include cervical swabs and amniotic fluid, placenta, and urine samples. Urine is least dangerous sample in pregnant women.

HPV is a DNA virus and causes reproductive tract infection in men and women. The relationship between the genome of this virus and cervical cancer was proved years ago; however, few studies have investigated the association between HPV infection and spontaneous abortion, which have shown non-significant associations [2, 3].

Similar to mycoplasma and chlamydia, the samples used for detection of HPV include swab specimens sampled from the cervix and urine.

In the present study, we decided to investigate three major causes of sexually transmitted disease (STDs) that also cause miscarriage in the first trimester in a number of pregnant women. Since cervical swabbing is invasive and dangerous and is not accepted by pregnant women, early urine specimens were used in this study.

## Methods

Considering a precision of 7% to detect the difference and a prevalence of 2.8% for *C. trachomatis*, 10% for *M. genitalium*, and 8.4% for HPV, the sample size was estimated 119, 61, and 66, respectively. Finally, the largest sample size was considered for the study by this formula:

$${\text{n}}^{\prime} = \frac{{{\text{NZ}}^{2} {\text{P}}(1 - {\text{P}})}}{{{\text{d}}^{2} ({\text{N}} - 1) + {\text{Z}}^{2} {\text{P}}(1 - {\text{P}})}}.$$

In this study, urine samples were collected from 119 pregnant women who visited Almahdi Clinic Center in south of Tehran for routine first-trimester screening (12–14 weeks gestation). Almahdi Clinic is under the supervision of the Tehran University of Medical Sciences. The sampling duration time was October 2016 to August 2017. Approvals of the Ethics Committees of all patients participating in the study were obtained (IR.TUMS.REC.1395-2322) The subjects first completed a questionnaire and gave informed consent. Then, urine specimens were collected. First void urine is the best urine sampling method for PCR to detect *C. trachomatis*, *M. genitalium*, and HPV. The subjects were requested no to pass urine for 4 h before sampling and then their first-catch urine was collected in sterile containers. About 10 ml of the sample was centrifuged at 3000×*g* for 20 min and 1–2 ml of the sediment was transferred to sterile microfuges and stored at − 20 °C until analysis.

DNA extraction was conducted using A101211 kits imported by Pars Tous Biotechnology Company. After extraction, DNA samples were stored at − 70 °C until PCR was done.

The following commercial kits, imported by Pars Tous Biotechnology, were used for PCR.

HPV PCR kit with code number A101032.

This PCR amplification kit includes all necessary PCR amplification reagents, capable of amplifying a wide spectrum of HPV types and a house keeping gene as internal control. The most well-known and clinically important HPV are detectable with this kit, including type 6, 11, 16, 18, 26, 31, 32, 33, 34, 35, 42, 45, 51, 52, 53, 55, 56, 58, 59, 61, 66, 67, 68, 69, 70, 73, PAP 155, PAP291, X4. Viral DNA present in positive samples is specifically amplified by using primers complementary to viral sequences.

*Mycoplasma genitalium* PCR kit with code number A101112. This PCR amplification kit includes all necessary PCR amplification reagents with an exception of mineral oil. The *M. genitalium* PCR kit is a qualitative analysis test, using a DNA amplification technique for direct detection of *M. genitalium* DNA. Bacterial DNA present in positive samples is specifically amplified by using primers complementary to bacterial sequences. Each kit offered Pars Tous’s optimal buffer system which will enhance amplification specificity with a detection limit of ~ 2 copies per reaction.

*Chlamydia trachomatis* nested PCR kit with code number A101122 is a qualitative analysis test, using a DNA amplification technique for direct detection of *C. trachomatis* DNA. Bacterial DNA present in positive samples is specifically amplified by using primers complementary to bacterial sequences. Each kit offered Pars Tous’s optimal buffer system which will enhance amplification specificity.

## Results

In this study, urine samples were collected from 119 pregnant women with mean age 29 years old who visited health centers for routine first-trimester screening (12–14 weeks gestation). The subjects first completed a questionnaire and gave informed consent. Then, urine specimens were collected and have detected *C. trachomatis*, *M. genitalium*, and HPV with PCR method.

The frequency distribution of abortion, preterm labor, multiple pregnancies history, number of pregnancies and methods of contraception in pregnant women studied are shown in Tables 1 and 2, respectively.

**Table 1:** Frequency distribution of abortion, preterm labor and multiple pregnancies history in pregnant women

Variable	Abortion history			Premature delivery		Multiple pregnancy		Total
	Non	One	More	No	Yes	No	Yes	
Number (%)	88 (73.95)	25 (21.01)	6 (5.04)	116 (97.48)	3 (2.52)	116 (97.48)	3 (2.52)	119

**Table 2:** Frequency distribution of variables, number of pregnancies and methods of contraception in pregnant women studied

Variables	Number (%)
Pregnancy number	
1	35 (29.41)
2	47 (39.50)
3	28 (23.53)
4	7 (5.88)
5	2 (1.68)
Pregnancy prevention method	
Natural	85 (71.43)
Condom	12 (10.08)
LD	12 (10.08)
IUD	8 (6.72)
Injection	2 (1.68)

Statistical analyses showed no significant relationship between the variables assessed in the questionnaire, including history of preterm delivery, number of pregnancies, history of multiple pregnancy, contraception method and urinary isolation of chlamydia and mycoplasma.

The number of positive *M. genitalium*, *C. trachomatis* and HPV in urine samples with PCR method is shown in Table 3.

**Table 3:** The urinary isolation of *M. genitalium*, *C. trachomatis* and HPV in pregnant women by PCR method

Microorganism isolated	Positive (%)	Negative (%)	Total
*M. genitalium*	21 (17.65)	98 (82.35)	119
*C. trachomatis*	8 (6.72)	111 (93.28)	119
HPV	0 (0)	119 (100)	119

The statistical analysis between urinary isolation of *C. trachomatis* and *M. genitalium* and miscarriage is shown in Tables 4 and 5, respectively.

**Table 4:** The statistical analysis between urinary isolation of *C. trachomatis* and miscarriage in pregnant women

Variables	Chlamydia infection		
Abortion	Positive	Negative	P-value
	Number (%)	Number (%)	
No	6 (75)	82 (73.87)	
Yes	2 (25)	29 (26.13)	0.93

**Table 5:** The statistical analysis between urinary isolation of *M. genitalium* and miscarriage in pregnant women

Variables	Mycoplasma infection		
Abortion	Positive	Negative	P-value
	Number (%)	Number (%)	
No	15 (71.43)	73 (74.49)	
Yes	6 (28.57)	25 (25.51)	0.80

According to Table 4, we found no significant association between urinary isolation of *C. trachomatis* and miscarriage (P = 0.93) and according to Table 5, we found no significant association between urinary isolation of *M. genitalium* and miscarriage (P = 0.80). Regarding HPV, since all urine samples were PCR-negative, comparison was not possible.

According to the present study, chlamydia was isolated from the urine samples of 6.72% of the pregnant women who underwent first-trimester screening in health centers using PCR. Previous studies reported a mean chlamydia isolation rate of 3% from urine specimens collected from pregnant women in general. T-test showed no significant difference between the two groups (P = 0.10).

In our study, the mycoplasma isolation rate was 17.65% using PCR. Previous studies reported a mean mycoplasma isolation rate of 10% from urine specimens collected from pregnant women in general. T-test showed a significant difference between the two groups (P = 0.03).

**Fig. 1:**
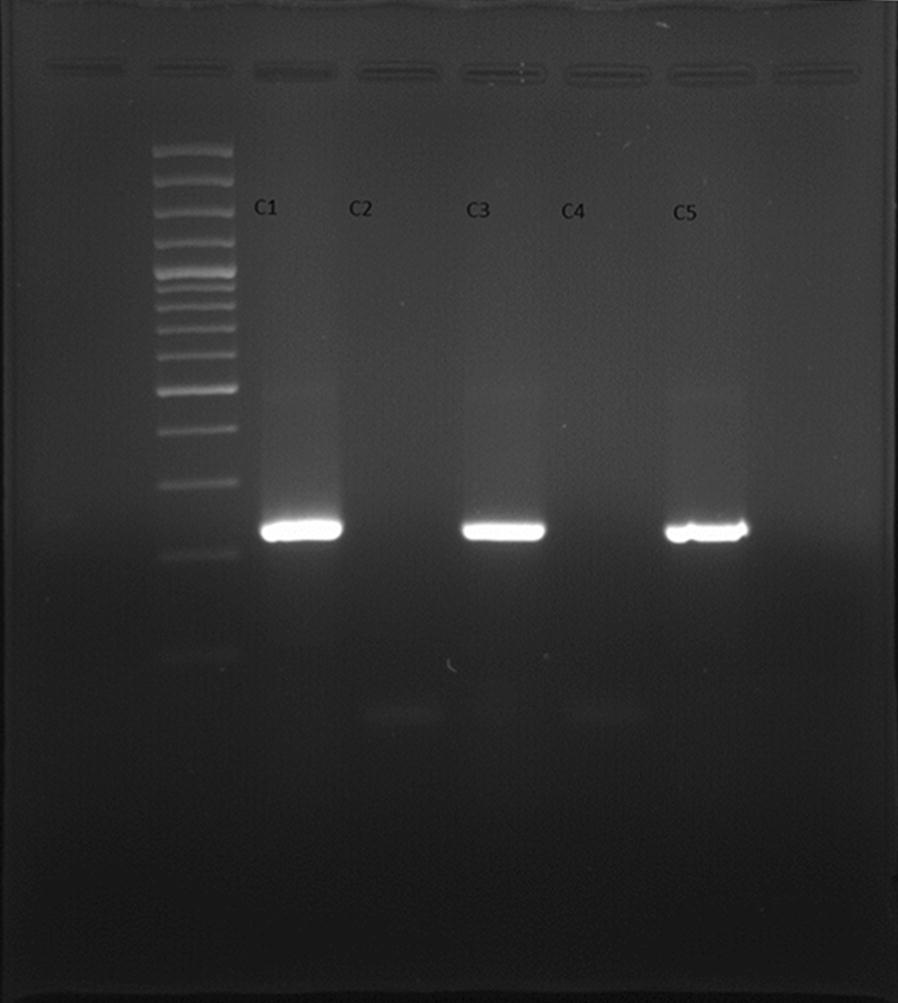
Gel electrophoresis of PCR products of *Chlamydia trachomatis* (*C1* control positive, *C2* control negative, *C3*–*5* urine samples)

**Fig. 2:**
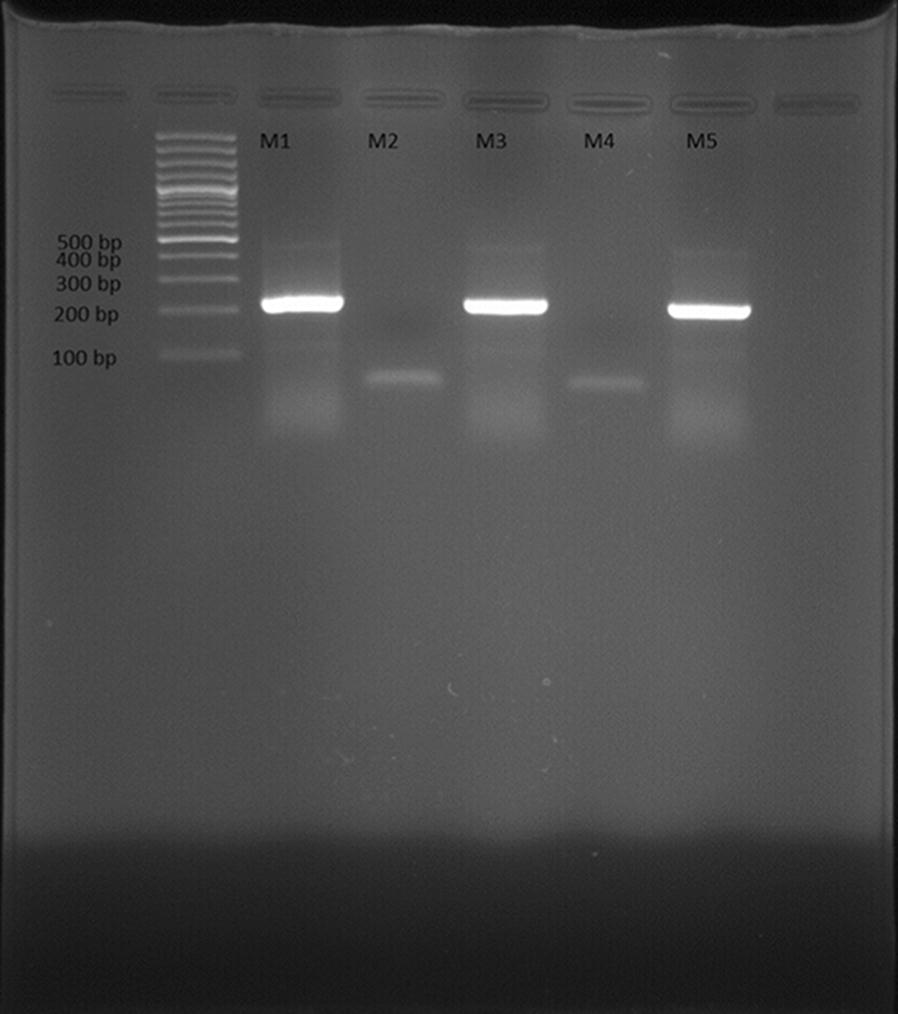
Gel electrophoresis of PCR products of *Mycoplasma genitalium* (*M1* control positive, *M2* control negative, *M3*–*5* urine samples)

The pictures gel electrophoresis of PCR products of *C. trachomatis* and *M. genitalium* is shown in Figs. 1 and 2, respectively

**Fig. 3:**
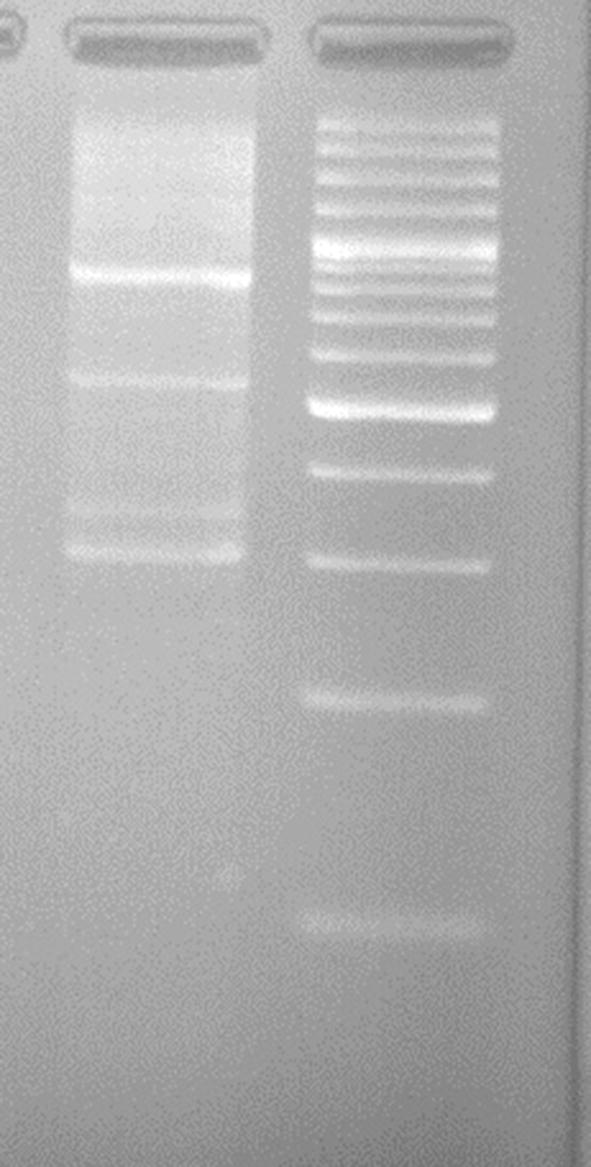
DNA quality

Figure 3 is about the DNA quality.

## Discussion

Miscarriage and preterm delivery are the most important challenges of pregnancy. Different bacterial and viral infection may cause miscarriage and preterm delivery [4]. Among bacterial factors, *M. genitalium* and *C. trachomatis* have the most important role and HPV is the leading viral factor in this regard [1].

All the above agents are colonized in the vaginal either symptomatically or asymptomatically. If symptomatic, the person seeks treatment. What is important is a woman in whom these agents are colonized in the vagina and the patient is asymptomatic. These women are at increased risk of preterm delivery or miscarriage if they become pregnant [1, 2, 5]

According to a study in Samoa, 239 women aged 18–29 years were evaluated for *C. trachomatis* infection using PCR. The results showed that 36% of the women were infected that were mostly 18–24 years. Urine samples were used in this study [6]. A retrospective study on 590 women between 2010 and 2014 showed that 11 and 6.9% of the women were infected with HPV and *C. trachomatis*, respectively [7]. Researchers in Switzerland compared 146 pregnant women with preterm delivery with 261 women with term delivery. The results of PCR showed a significantly higher rate of *C. trachomatis* infection in women with preterm delivery [8]. In the USA, about 3–6% of the women below 25 years who look healthy have *C. trachomatis* infection. In general, the percentage of *C. trachomatis* infection is different in different countries; for example, 3.9% in England, 6% in Finland, 17% in France, 9% in Poland, and 6.5–22% in Iran. In a study in 2016, *C. trachomatis* DNA was detected on the endocervical swabs of 17.43% of the subjects [9]. A similar study in Iran showed the colonization rate of *C. trachomatis* is 2.8% in pregnant women. The rate is reported to be about 4.8% in the urine of pregnant women with a history of miscarriage or infertility [10, 11].

Numerous studies have investigated *M. genitalium*. The colonization rate of mycoplasma is 12–50% in pregnancy but the prevalence of mycoplasma is even higher in women with a history of miscarriage. A colonization rate of about 84% was reported in a study in Malaysia.

A similar study in China showed that the colonization rate of *Mycoplasma hominis* was about 27.6 and 10% in women with a history of miscarriage and normal women, respectively. According to a study in Iran, mycoplasma is colonized in 10% of the pregnant women [10]. A similar study showed a colonization rate of 21–45% for mycoplasma in women [12].

As for HPV, studies have shown that the DNA of this virus can be detected in the vaginal swabs of 30–45% of women using PCR. In another study, HPV was isolated from vaginal swabs of 5.5–65% of the subjects, depending on the geographical region, using PCR. In a study in Poland, HPV was isolated from the vaginal specimens of 35.14% of pregnant women using PCR [13, 14]. Another study reported an isolation rate of 36%. A study in Brazil, HPV DNA was detected in the vaginal samples of 25.3% of pregnant women and 13% of non-pregnant women using PCR, which showed a significant difference [14]. The results of a study in Iran showed that of 2577 healthy women, HPV DNA was isolated from 8.4% of vaginal specimens using PCR [15].

The results of a study by Ducancelle et al. showed that HPV was isolated from the 42 and 49% of the urine and cervical discharge samples of 230 women, respectively. In this study, real time PCR was used to detect HPV. The women participating in this study were not pregnant, so there was no risk of miscarriage. The isolation rate of the two sampling methods had a significant correlation [16]. In a similar study, the correlation of HPV isolation from cervical discharge specimens (45%) and urine samples (37%) using real time PCR was about (93%) [17]. Tamim et al. assessed *C. trachomatis* and HPV in cervical discharge and urine specimens (2–4 h after urinary retention) using PCR [18].

Vanessa et al. investigated the prevalence of mycoplasma in the urine samples of 82 women with a history of miscarriage and 34 pregnant women 22 weeks gestation and reported similar results [19]. Jensen et al. reported that urine specimens are better than urogenital swabs for detection of mycoplasma and chlamydia using PCR [20].

About antibiotic treatment of genital infections with *C. trachomatis* fortunately investigations has shown the clinical strains of *C. trachomatis* with macrolide or fluoroquinolone resistance would be uncommon, and azithromycin or fluoroquinolone regimens could be recommended as treatments for chlamydial infections [21].

Although in genital infections caused by *M. genitalium* based on Anderson et al. investigation, the clinical observation of increasing treatment failure indicating antibiotic resistance has been confirmed by molecular testing. Fluoroquinolone mutation screening was performed on 86 (74.8%) of the 115 samples, of which 20 (23.3%) samples had a mutation or mutations associated with increased resistance. This study shows the increasing antibiotic resistance and the need for appropriate guidelines to treat at-risk patients [22].

The most common sampling method is vaginal and cervical sampling to detect the above bacterial and viral agents, which required the use of speculum. Since this sampling method is invasive and may cause miscarriage, less invasive methods should be used. In recent years, the results of studies isolating these bacterial and viral agents from first void urine have been promising. In this method, the patient should not void for at least 4 h, and then first-catch urine is collected a sterile container for urine culture.

In our study, first void urine was used to detect the above bacterial and viral agents that play a role in miscarriage and preterm delivery. One hundred and nineteen pregnant women (11–14 weeks gestation) who underwent first-trimester screening in health centers were recruited. After completing the questionnaire and obtaining informed consent, the subjects were given urine collection containers to collect their urine. Urine samples were centrifuged for 20 min and DNA was extracted from the sediment. PCR was done on the samples to detect *M. genitalium*, *C. trachomatis*, and HPV. According to the results, chlamydia was isolated from the urine specimens of 6.78% of the pregnant women. Previous studies reported a mean chlamydial isolation rate of 3% from urine specimens collected from pregnant women in general. T-test showed no significant difference between the two groups (P = 0.10). According to the above results, the isolation rate of chlamydia from urine specimens of pregnant women was more than two times higher than vaginal and cervical discharge specimens because previous studies showed an isolation rate of 3% for *C. trachomatis* from vaginal and cervical discharge specimens. Therefore, urine is an appropriate sample for detection of *C. trachomatis* because its collection is non-invasive and offers a higher isolation rate.

According to our results, the mycoplasma isolation rate was 17.8% using PCR. Previous studies reported a mean mycoplasma isolation rate of 10% from urine specimens collected from pregnant women in general. T-test showed a significant difference between the two groups (P = 0.03).

The 10% prevalence of mycoplasma in the pregnant women was based on cervical or vaginal specimens, which is an invasive sampling method in pregnant women and is not well accepted. We used urine samples in our study and found a higher isolation rate than vaginal and cervical discharge specimens. Therefore, first void urine may yield better results.

We found no significant relationship between the isolation rate of mycoplasma and chlamydia using PCR and the history of miscarriage. Since HPV was not isolated from any of the urine specimens, statistical analysis was not possible. HPV was isolated from 8.4% of vaginal and cervical discharge specimens of pregnant women; therefore, it may be concluded that urine is not a good sample for HPV detection and cervical or vaginal specimens should be used to detect these viral agent.

All 119 women were followed up until delivery. Three cases of preterm delivery were noted, of whom two were positive for mycoplasma and one was positive for both mycoplasma and chlamydia. Statistical analysis showed a significant difference in preterm delivery between women who were positive for one of the above agents and women who were negative.

In general, the aim of the present study was to isolate three important causes of septic abortion, i.e., *C. trachomatis*, *M. genitalium*, and HPV, from urine specimens of pregnant women in the first trimester. According to the results, first void may be an appropriate sample for detection of *C. trachomatis* and *M. genitalium*; however, urine is not a good sample for HPV isolation and vaginal or cervical discharge specimens should be used instead. Regarding mycoplasma, urine specimens are even better than vaginal and cervical specimens considering the higher isolation rate.

## Abbreviations

*M. genitalium*: *Mycoplasma genitalium*
*C. trachomatis*: *Chlamydia trachomatis*
HPV: Human papilloma virus
STD: Sexually transmitted diseases
